# Preliminary Study on Circulating REG3α and Its Associations with Vitamin D Supplementation and Inflammatory Biomarkers in Adults with Overweight and Obesity

**DOI:** 10.3390/cimb47120970

**Published:** 2025-11-21

**Authors:** Theocharis Koufakis, Dimitrios Kouroupis, Areti Kourti, Paraskevi Karalazou, Katerina Thisiadou, Ioannis Georgiadis, Omar Mustafa, Giuseppe Maltese, Luca Busetto, Djordje S. Popovic, Olga Giouleme, Kalliopi Kotsa, Michael Doumas, Kali Makedou

**Affiliations:** 1 Second Propaedeutic Department of Internal Medicine, School of Medicine, Aristotle University of Thessaloniki, Hippokration Hospital, 54642 Thessaloniki, Greece; dimcour841@gmail.com (D.K.); giouleme@auth.gr (O.G.); michalisdoumas@yahoo.co.uk (M.D.); 2 Laboratory of Biochemistry, School of Medicine, Aristotle University of Thessaloniki, AHEPA University Hospital, 54124 Thessaloniki, Greece; aretikourti@auth.gr (A.K.); vivikarala@gmail.com (P.K.); thisiadou@yahoo.gr (K.T.); kalimakedou@gmail.com (K.M.); 3 National Primary Health Care Network, 61200 Polykastron, Greece; ioannis.georgiadis@med.uni-giessen.de; 4 Department of Diabetes, King’s College Hospital NHS Foundation Trust, London SE5 9RS, UK; omarmustafa@nhs.net; 5 King’s College, London WC2R 2LS, UK; 6 Department of Diabetes and Endocrinology, Epsom & St Helier University Hospitals, Epsom KT18 7EG, UK; giuseppe.maltese@kcl.ac.uk; 7 Unit for Metabolic Medicine, Cardiovascular Division, Faculty of Life Sciences & Medicine, King’s College, London WC2R 2LS, UK; 8 Department of Medicine, University of Padova, 35122 Padova, Italy; luca.busetto@unipd.it; 9 Clinic for Endocrinology, Diabetes and Metabolic Disorders, Clinical Centre of Vojvodina, Medical Faculty, University of Novi Sad, 21000 Novi Sad, Serbia; pitstop021@gmail.com; 10 Division of Endocrinology and Metabolism and Diabetes Center, First Department of Internal Medicine, Medical School, Aristotle University of Thessaloniki, AHEPA University Hospital, 54636 Thessaloniki, Greece; kkalli@auth.gr

**Keywords:** REG3α, obesity, vitamin D, inflammation, gut dysbiosis, antimicrobial peptides, mucosal immunity

## Abstract

Objective: Obesity is characterized by chronic inflammation and gut dysbiosis, yet circulating markers reflecting intestinal immune activation remain limited. Regenerating islet-derived protein 3 alpha (REG3α), an antimicrobial peptide secreted by intestinal Paneth cells, plays a pivotal role in mucosal defense and mirrors microbial–epithelial interactions. In this cross-sectional study, we aimed to examine circulating REG3α levels in infection-free adults with obesity, overweight, and normal weight, and to explore their associations with vitamin D supplementation and biomarkers of inflammation and dysbiosis. Methods: Sixty-nine participants were stratified into control, overweight, and obesity groups. Serum REG3α, interleukin-6 (IL-6), β-defensin-2, high-sensitivity c-reactive protein, ferritin, and presepsin were assessed. Vitamin D status and supplementation history were recorded. Multivariable linear regression, principal component analysis (PCA), and bootstrap mediation models were applied to explore associations and potential indirect effects. Results: REG3α concentrations were higher in overweight and obesity compared to controls (646 ± 217 vs. 521 ± 311 ng/mL); however, the difference was not significant (*p* = 0.15). Vitamin D supplementation was inversely associated with REG3α (*p* = 0.06), and this effect appeared weaker in obesity. REG3α correlated positively with IL-6 (ρ = 0.28) and β-defensin-2 (ρ = 0.43). PCA revealed a shared inflammatory–mucosal activation component that predicted REG3α levels. Exploratory mediation suggested a partial indirect effect of vitamin D via reduced inflammatory activity. Conclusions: Circulating REG3α shows associations with indicators of inflammation and vitamin D supplementation in individuals with overweight and obesity. Although differences between groups did not reach statistical significance, the observed trends suggest possible links between mucosal immune activity and metabolic status. These exploratory results warrant validation in larger, longitudinal studies before any biomarker role can be established.

## 1. Introduction

Obesity is recognized as a chronic inflammatory condition that gradually disrupts immune and metabolic homeostasis [1]. The continuous release of cytokines and adipokines from hypertrophic fat depots sustains a low-grade systemic inflammation that affects multiple organs [2]. Among the mechanisms linking adiposity to inflammation, gut dysbiosis plays an increasingly acknowledged role. Dysbiosis promotes intestinal permeability and bacterial translocation, exposing the host to microbial products that stimulate immune pathways and maintain inflammatory activity [3]. In this context, the gut barrier becomes both a target and a source of inflammation, establishing a vicious cycle that underlies many obesity-related metabolic disturbances.

Among the molecules involved in mucosal defense, the antimicrobial peptide regenerating islet-derived protein 3 alpha (REG3α) has attracted growing interest. REG3α is secreted primarily by intestinal Paneth cells and, to a lesser extent, by enterocytes, in response to bacterial colonization and cytokines such as interleukin (IL)-22 [4]. It acts by binding peptidoglycan and selectively killing Gram-positive bacteria, thereby helping to maintain a spatial segregation between the microbiota and the epithelium [5]. Beyond local effects, circulating REG3α reflects intestinal barrier activity and microbial translocation. Elevated blood levels have been reported in conditions like inflammatory bowel disease, sepsis, and graft-versus-host disease—all characterized by barrier dysfunction and systemic immune activation [6,7]. Despite these connections, the role of REG3α in obesity, where low-grade inflammation and microbial imbalance coexist, remains largely unexplored.

Understanding REG3α within the inflammatory landscape of obesity requires examining accompanying immune and mucosal markers. IL-6 represents a major cytokine driving hepatic acute-phase responses and promoting insulin resistance [8]. β-defensin-2 is another antimicrobial peptide released at mucosal surfaces, signaling epithelial engagement [9]. C-reactive protein, though systemic, remains a reliable marker of subclinical inflammation and cardiometabolic risk [10]. Presepsin reflects monocyte activation by bacterial components, while ferritin behaves as an acute-phase reactant in chronic inflammation [11]. Together, these biomarkers outline different layers of immune activation—from microbial sensing to systemic inflammation—within which REG3α may serve as an integrative signal.

Vitamin D is increasingly recognized as a modulator of immune and epithelial function. Through activation of the vitamin D receptor, it influences cytokine balance, macrophage activity, and epithelial integrity [12]. Deficiency, highly prevalent in obesity, has been associated with dysregulated inflammation and impaired antimicrobial peptide synthesis, including cathelicidins and defensins [13]. The link between vitamin D and REG3α remains speculative, yet plausible, since both are responsive to epithelial immune cues [14]. Vitamin D supplementation could, therefore, modify REG3α expression indirectly by attenuating inflammation or directly through epithelial gene regulation.

Emerging evidence suggests that metabolic inflammation and intestinal barrier dysfunction may not act in parallel but are causally interconnected. Experimental models have shown that altered gut permeability allows microbial metabolites, such as lipopolysaccharides and short-chain fatty acids, to reach systemic circulation, triggering inflammatory signaling cascades that perpetuate insulin resistance and adipose tissue remodeling [15]. These findings have led to the concept of “metabolic endotoxemia” where intestinal integrity plays a regulatory role in metabolic homeostasis. In this context, identifying circulating molecules that capture this axis could be of particular value. REG3α, due to its selective activity against Gram-positive bacteria and role in maintaining spatial segregation between luminal microbes and host epithelium, may serve as a measurable proxy for the functional state of the intestinal barrier [16]. Investigating its behavior in obesity could therefore provide insight into the early immune adaptations that accompany metabolic dysfunction.

The present study aimed to characterize circulating REG3α concentrations in adults with obesity, overweight, and normal-weight controls, and to examine their associations with vitamin D status, supplementation, and biomarkers of inflammation and dysbiosis. A secondary goal was to integrate these markers into composite indices to explore broader inflammatory patterns. By addressing this gap, our work seeks to determine whether REG3α could serve as a biomarker of mucosal immune activity and microbial imbalance in metabolic conditions. To our knowledge, this is the first study to evaluate the role of REG3α in obesity in humans.

## 2. Materials and Methods

### 2.1. Study Population

This cross-sectional study included adults aged ≥18 years with overweight and obesity [Body Mass Index (BMI) 25.0–29.9, and ≥30 kg/m^2^, respectively] who were recruited from outpatient obesity clinics. Hospital employees served as normal-weight controls (BMI 18.5–24.9 kg/m^2^). Participants were consecutively enrolled between January and May 2024 from the Obesity Clinics of Hippokration and AHEPA University Hospitals (Thessaloniki, Greece). Inclusion criteria required stable weight during the previous three months and absence of acute infection. Exclusion criteria included diabetes, history of autoimmune or malignant disease, active smoking (participants who had stopped smoking for at least six months were eligible), chronic kidney disease with an estimated glomerular filtration rate <60 mL/min/1.73 m^2^, hepatic impairment defined as a known liver disorder or aminotransferase levels exceeding twice the upper limit of normal, recent surgery within the previous three months or other severe inflammatory condition (such as pancreatitis, burns, etc.), and use of corticosteroids or immunosuppressive drugs. This was a pilot exploratory study; therefore, no formal sample size calculation was performed. The sample size reflects the number of eligible participants meeting the inclusion criteria during the recruitment period.

### 2.2. Clinical Evaluation and Laboratory Methods

Demographic, anthropometric, and laboratory information were obtained from each participant. Anthropometric evaluation comprised measurements of body weight and height. Height was measured to the nearest millimeter using a Holtain wall-mounted stadiometer, and body weight was determined with a calibrated computerized digital scale (K-Tron P1-SR, Onrion LLC, Bergenfield, NJ, USA) accurate to 0.1 kg. All assessments were performed with participants barefoot and wearing light clothing. BMI was calculated as weight (kg) divided by height squared (m^2^). Although direct body composition measurements were not available, BMI remains the most validated and widely used indicator of adiposity in epidemiological and clinical studies, facilitating comparability with previous research. To ensure accuracy, a second examiner independently verified all anthropometric measurements.

Information regarding vitamin D supplementation was obtained through structured interviews and confirmed, when available, by medical records. Participants were categorized as supplemented if they were currently taking vitamin D, either as prescribed by their general practitioner or purchased over the counter. The duration of supplementation (in months) was recorded. Because formulations and doses varied across individuals, supplementation status was analyzed as a binary variable (yes/no) in the main models, with duration considered as a continuous covariate in exploratory analyses.

Venous blood samples were collected after a 12 h overnight fast. Following collection, samples were promptly centrifuged, and serum aliquots were stored at −20 °C until further analysis. Serum ferritin and IL-6 concentrations were determined using electrochemiluminescence immunoassays on the ELECSYS platform (Roche Diagnostics, Mannheim, Germany). High-sensitivity C-reactive protein (hs-CRP) was measured by a particle-enhanced immunoturbidimetric assay on the COBAS Pure analyzer (Roche Diagnostics, Mannheim, Germany). Serum presepsin concentrations were quantified with a commercial kit, based on sandwich enzyme-linked immune-sorbent assay (ELISA) technology (Fine Biotech Co., Ltd., Wuhan, China), with a detection range of 0.156–10 ng/mL and with intra-assay and inter-assay variation of 4.93% and 4.93%, respectively. β-defensin 2 serum levels were determined using a sandwich ELISA based commercial kit of the same company, with a detection range of 15.625–1000 pg/mL and with intra-assay and inter-assay variation of 5.41% and 5.25%, respectively. REG3α serum levels were also determined with a sandwich ELISA kit of the same company, with a detection range of 15.625–1000 pg/mL and with intra-assay and inter-assay variation of 5.13% and 5.15%, respectively. Vitamin D status was assessed by measuring 25-hydroxy-vitamin D [25(OH)D] using an electrochemiluminescence method on e801 immunochemistry module of COBAS 8000 system (Roche Diagnostics, Mannheim, Germany). All biochemical measurements were performed in the same accredited central laboratory to ensure analytical consistency and quality control.

### 2.3. Statistical Analysis

Continuous data are expressed as mean ± standard deviation or median (interquartile range), and categorical variables as counts and percentages. Visual inspection of the REG3α distribution (Figure S1), together with the Shapiro–Wilk test (*p* < 0.05), confirmed deviation from normality. Consequently, non-parametric tests (Kruskal–Wallis and Mann–Whitney U) were applied for group comparisons, while normally distributed variables were analyzed using one-way ANOVA. Categorical variables were compared using the χ^2^ test. Linear regression models were constructed with circulating REG3α as the dependent variable, adjusting for BMI group, age, and sex. Additional models included vitamin D supplementation status, duration of supplementation, history of cardiovascular disease (CVD), and biomarker covariates. Robust standard errors (HC3) were applied to account for heteroskedasticity.

Correlations between continuous variables were assessed using Spearman’s ρ. Principal component analysis (PCA) was performed on inflammatory and mucosal biomarkers (IL-6, β-defensin-2, hs-CRP, ferritin, and presepsin) to summarize shared variability and derive a composite index (PCA1). The purpose of PCA was to reduce dimensionality and capture common inflammatory–mucosal activity rather than analyzing each biomarker separately. The first principal component (PCA1) was retained, as it explained the largest portion of variance and represented the dominant biological pattern. Given the modest sample size and exploratory nature of this study, PCA and mediation analyses were performed primarily as hypothesis-generating tools to identify potential biological patterns rather than to establish causality or robust mechanistic pathways. The results should therefore be interpreted with caution.

To further illustrate associations between REG3α, adiposity, and vitamin D supplementation, exploratory logistic regression and Fisher’s exact tests were conducted. REG3α concentrations were dichotomized at the sample median to define “high” and “low” REG3α categories. Odds ratios (ORs) and corresponding 95% confidence intervals (CIs) were estimated to compare the likelihood of elevated REG3α between supplemented and non-supplemented participants within BMI strata. Finally, exploratory mediation analyses examined whether the relationship between vitamin D supplementation and REG3α could be partially explained by PCA1, adjusting for age, sex, and adiposity. Indirect effects were tested with non-parametric bootstrap resampling (5000 iterations) to obtain bias-corrected 95% confidence intervals. Statistical analyses were conducted using R software (version 4.3.1; R Foundation for Statistical Computing, Vienna, Austria) and significance was set at *p* < 0.05.

### 2.4. Ethical Considerations

The study was approved by the Bioethics Committee of the Aristotle University of Thessaloniki (approval code: 6.233; approval date: 29 July 2020) and conducted in accordance with the Declaration of Helsinki. All participants provided written informed consent prior to participation.

## 3. Results

### 3.1. Participant Characteristics and Biomarker Profiles

Sixty-nine participants were included: 18 controls, 29 persons with overweight, and 22 individuals with obesity. Mean age was comparable between groups, and women predominated slightly. Serum 25(OH)D concentrations were lower in overweight and obesity but without significant difference. The prevalence of vitamin D supplementation differed modestly among groups, reported in 35% of controls, 32.4% of participants with overweight, and 33.3% of those with obesity (*p* = 0.98). The mean duration of supplementation among users was 8.4 ± 3.7 months, with no significant difference across categories (*p* = 0.42). Inflammatory biomarkers showed the expected directional trends, with higher mean concentrations of IL-6, β-defensin-2, ferritin, and hs-CRP in the obesity group compared with controls; however, these differences were not significant (Table 1).

**Figure 1:**
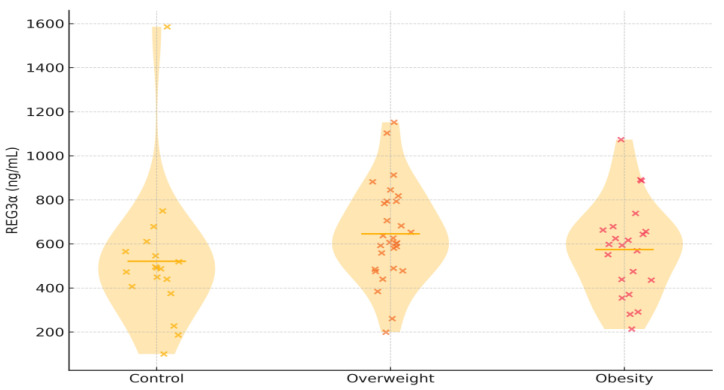
Violin plots depict the distribution of serum REG3α concentrations among the three study groups. Each x represents an individual participant. Abbreviations: REG3α—regenerating islet-derived protein 3 alpha.

REG3α concentrations were numerically highest in individuals with overweight, intermediate in those with obesity, and lowest in controls (mean 646.03 ± 217.09 ng/mL, 574.78 ± 212.07 ng/mL, and 521.49 ± 311.27 ng/mL, respectively), although the overall difference between groups did not reach statistical significance (*p* = 0.226). Unadjusted linear regression indicated increases of +53 ng/mL in obesity and +125 ng/mL in overweight compared with controls. Adjusting for age and sex did not change these estimates. Age displayed a weak positive trend, whereas sex had no measurable effect. Combining overweight and obesity in one group yielded similar results. Figure 1 illustrates the overall distribution, showing substantial overlap among groups but a visible upward shift in the overweight category. Exploratory pairwise comparisons are provided in Table S1.

### 3.2. REG3α Association with Vitamin D Supplementation

Vitamin D supplementation was associated with lower REG3α concentrations independently of age, sex, and adiposity. In adjusted models, this effect approached significance (OR = 0.31; 95% CI: 0.09–1.08; *p* = 0.06) and appeared stronger in controls than in overweight or obesity. Adjustment for supplementation duration and CVD history did not materially alter the association, indicating that differences in the length of vitamin D use within the present cohort were not sufficient to modify REG3α concentrations.

Predicted REG3α levels were lowest among participants receiving vitamin D supplementation and highest among those without supplementation and with higher BMI. Consistent with this trend, vitamin D supplementation was associated with substantially lower odds of elevated REG3α in participants with higher BMI (unadjusted OR = 0.19, 95% CI: 0.06–0.64, *p* = 0.0066). To further explore whether this association persisted after accounting for inflammatory activity and age, a multivariable logistic regression model was constructed including vitamin D supplementation, age, IL-6, and β-defensin-2 as covariates (Table 2).

Vitamin D supplementation remained an independent predictor of lower odds of elevated REG3α (adjusted OR = 0.26, 95% CI: 0.07–0.95, *p* = 0.041). None of the other covariates were significant. This finding reinforces that the inverse association between vitamin D use and REG3α is not merely a reflection of systemic inflammation but may indicate a more direct immunomodulatory effect.

### 3.3. Correlation of REG3α with Inflammatory Biomarkers and Exploratory Mechanistic Analyses

REG3α correlated positively with β-defensin-2 (ρ = 0.43, *p* = 0.0002) and IL-6 (ρ = 0.28, *p* = 0.022), while hs-CRP showed a weaker trend (*p* = 0.06). Ferritin, presepsin, and serum 25(OH)D were not significantly associated (Table 3 and Figure S2). In the multivariable linear regression model including IL-6 and β-defensin-2, both biomarkers remained independent predictors of REG3α, whereas vitamin D supplementation retained a negative coefficient (*p* = 0.07).

PCA of inflammatory and mucosal biomarkers identified one dominant component (PCA1), explaining approximately 50% of the shared variance, with the highest loadings observed for IL-6 and β-defensin-2. PCA1 correlated significantly with REG3α (ρ = 0.37, *p* = 0.006), suggesting that REG3α reflects a common inflammatory–mucosal activation axis rather than isolated biomarker variation (Figure S3).

To further explore potential mechanisms linking vitamin D and REG3α, a mediation analysis was performed using PCA1 as a latent mediator. The analysis using non-parametric bootstrap resampling (5000 iterations) suggested a possible indirect effect of vitamin D supplementation through PCA1 (β_indirect = −18.6; 95% bootstrap CI −42.1 to +2.4; *p* = 0.08), indicating that part of the association between vitamin D supplementation and REG3α may operate through modulation of inflammatory–mucosal activity. Sensitivity analyses excluding individuals with CVD or extreme biomarker values yielded comparable results, supporting the robustness of these exploratory findings.

## 4. Discussion

The present study provides the first evidence that circulating REG3α levels vary in relation to adiposity and vitamin D supplementation in humans. REG3α concentrations were numerically, but not statistically, higher in individuals with overweight and obesity compared to controls, while vitamin D supplementation was associated with lower levels across models. Although these associations did not reach statistical significance, the direction and consistency of the observed trends across BMI groups and analytical models support their biological plausibility. The persistence of these patterns after adjustment for inflammatory markers suggests that REG3α may be linked to obesity-related immune activation, although the study’s limited sample size likely reduced statistical power to detect modest effects. Exploratory mediation analysis indicated that a minor component of the vitamin D–REG3α relationship might act indirectly through inflammatory–mucosal pathways. Together, these findings suggest that REG3α may participate in pathways of mucosal–systemic immune activation in obesity and could be responsive to vitamin D–related immune modulation. However, given the exploratory and cross-sectional nature of this study, these associations should be interpreted as preliminary and hypothesis-generating rather than indicative of a confirmed biomarker relationship.

REG3α plays a unique role at the interface of the intestinal epithelium and the microbiota, acting as a molecular mediator of host–microbe segregation and mucosal repair [17]. The trend toward higher REG3α levels in participants with overweight, followed by a modest decline in obesity, may represent a biphasic compensatory response. In early metabolic disturbance, the mucosal barrier likely remains reactive, producing higher levels of REG3α to counter microbial stress. With sustained inflammation, however, epithelial dysfunction and Paneth cell fatigue may blunt this defense, leading to relatively lower concentrations despite persistent immune activation. This pattern mirrors other innate mediators that rise transiently during mild stress and decline as chronic dysfunction develops [18]. Nonetheless, this interpretation should be viewed as hypothesis-generating rather than confirmatory, given the small sample size and cross-sectional design.

The inverse association with vitamin D supplementation further supports a regulatory link between epithelial integrity and immune activation. Vitamin D modulates cytokine signaling, strengthens barrier function, and influences antimicrobial peptide expression [19]. Lower REG3α levels among supplemented individuals may indicate reduced epithelial stress or microbial translocation, consistent with the anti-inflammatory effects of vitamin D [20]. The weaker association observed in obesity could reflect impaired vitamin D receptor signaling or chronic immune desensitization [21]. Although REG3α plays a protective role within the intestinal epithelium, its elevation in circulation is generally interpreted as a response to barrier stress or microbial stimulation. Thus, lower circulating REG3α concentrations in supplemented individuals likely reflect a reduced requirement for mucosal defense activation. Vitamin D improves epithelial integrity and tight-junction organization, which may lessen microbial translocation and thereby diminish the stimulus for REG3α secretion [22]. In this context, the inverse relationship between vitamin D supplementation and REG3α supports the notion that supplementation alleviates mucosal immune activation.

The positive correlations between REG3α, IL-6, and β-defensin-2 highlight its integration within a broader mucosal–inflammatory axis. IL-6 activates STAT3-dependent pathways that drive epithelial defense, while β-defensin-2 represents an epithelial effector with overlapping regulation [23,24]. By summarizing these relationships through PCA, this study demonstrated that REG3α parallels a composite inflammatory–mucosal activation pattern rather than any single marker. Beyond its biological interpretation, the present findings raise conceptual implications for how immune and metabolic systems interact. The parallel regulation of REG3α, IL-6, and β-defensin-2 suggests that obesity-related inflammation extends beyond adipose tissue, involving the intestinal epithelium as both a source and a target of immune signaling. This supports an emerging view of obesity as a disorder of tissue communication rather than inflammation confined to fat depots [25]. The observed modulation by vitamin D further emphasizes the potential for micronutrient status to reshape immune–epithelial crosstalk. From a translational perspective, REG3α could help identify individuals in an early “adaptive” phase of mucosal activation before overt metabolic disease develops. Such early markers might guide interventions aimed at restoring gut barrier function and immune balance through dietary, probiotic, or vitamin D–based approaches [26]. However, more studies are needed before any biomarker role can be established.

This study extends our group’s previous findings on endocrine–immune interactions in obesity to the mucosal frontier. In previous work, we reported that vitamin D status and supplementation influence inflammatory and metabolic profiles in individuals with obesity, highlighting an immunomodulatory and hormonal interplay [27]. In other studies, we demonstrated coordinated alterations in cytokines, adipokines, and antimicrobial peptides, revealing that immune dysregulation in obesity is not confined to adipose tissue but reflects a broader systemic activation [28,29]. The current analysis adds a new dimension by incorporating REG3α as an indicator of gut-related immune activity. The observed pattern of higher REG3α levels in overweight, their partial attenuation in obesity, and their inverse relationship with vitamin D supplementation together suggest that epithelial immune activation is an integral part of the inflammatory phenotype previously described. Recent evidence from independent research groups has further underscored the relevance of REG3 proteins in mucosal and metabolic regulation. Heazlewood et al. [30] demonstrated that mucin dysregulation and epithelial stress induce REG3 expression as part of a protective mucosal response in inflammatory conditions. Gonzalez et al. [31] showed that REG3α modulates glucose homeostasis and insulin resistance in obese diabetic mice, linking REG3α activity to metabolic inflammation. Additionally, Hlavaty et al. [32] reported that circulating REG3α reflects intestinal barrier function and inflammatory activity in clinical settings. Taken collectively, these data delineate a continuum from endocrine and systemic inflammation to mucosal immune dysregulation, reinforcing the concept that obesity represents a multisystem disorder of immune and barrier regulation rather than a purely metabolic disease [33].

Emerging evidence suggests that circulating REG3α concentrations may be influenced by factors that regulate mucosal homeostasis, including the gut microbiome and dietary composition. Alterations in gut microbial communities can modulate intestinal epithelial integrity and immune signaling, thereby affecting REG3α expression and release [34]. Dietary components such as fiber, polyphenols, and fat content are also known to impact microbial metabolites and mucosal inflammation, which could secondarily alter REG3α levels [35]. Considering these complex host–microbe–diet interactions provides a broader context for interpreting REG3α as a dynamic marker reflecting intestinal and systemic immune crosstalk. However, data on the association between REG3α and these factors remain very limited or non-existent, highlighting the need for further research to clarify these relationships.

Despite its exploratory design, this study presents several noteworthy strengths. First, it integrates mucosal and systemic biomarkers in a unified analytical framework, allowing REG3α to be interpreted within the broader context of inflammatory and epithelial signaling. Second, the inclusion of vitamin D supplementation and duration data adds a unique dimension, linking micronutrient status to immune–mucosal interactions in obesity—an area rarely explored in human studies. However, several limitations merit consideration. The sample size was modest, limiting power to detect between-group differences or interaction effects, particularly after adjustment for multiple covariates. This limitation may have contributed to the absence of statistically significant findings and to the borderline association observed with vitamin D supplementation. In addition, the relatively small and uneven group sizes may have increased variability within groups, further reducing statistical sensitivity. The use of multivariate techniques such as PCA and mediation models in a small dataset carries inherent statistical uncertainty. These analyses were conducted to generate hypotheses regarding potential pathways linking vitamin D, inflammation, and REG3α, rather than to imply mechanistic causality. An additional limitation of this study is the use of BMI rather than direct measures of body fat or body composition. While BMI does not distinguish between lean and fat mass, it remains the standard metric employed in the vast majority of epidemiological studies investigating obesity-related outcomes, ensuring comparability with previous findings. Dietary and microbiome factors were not measured but could influence results. In addition, physical activity represents another important determinant of mucosal and systemic inflammatory tone that was not assessed in the present study [36]. The lack of data on dietary habits, microbial composition, and activity level constitutes a source of residual confounding that should be addressed in future research integrating metabolic, microbial, and behavioral parameters. Although our results suggest that vitamin D supplementation was associated with lower circulating REG3α concentrations, this cross-sectional analysis cannot establish causality. The observed relationships should therefore be interpreted as descriptive and hypothesis-generating. Prospective and mechanistic studies are required to determine whether vitamin D supplementation directly modulates REG3α expression or whether both vary in parallel as part of broader immune–metabolic interactions.

## 5. Conclusions

This exploratory study examined circulating REG3α in relation to inflammation and vitamin D status across BMI categories. While REG3α concentrations did not differ significantly between normal-weight, overweight, and obesity groups, the peptide showed significant associations with vitamin D supplementation and with inflammatory biomarkers such as β-defensin-2 and IL-6, supporting a potential link between mucosal immune activity and metabolic inflammation. These findings suggest a biologically plausible, though preliminary, interplay between vitamin D—related immune modulation and REG3α expression; however, the present data do not establish causality or confirm a biomarker role. Given the modest sample size and cross-sectional design, the results should be viewed as hypothesis-generating and warrant confirmation in larger, longitudinal, and mechanistic studies.

## Figures and Tables

**Table 1:** Participant Characteristics by Group.

Variable	Control (n = 18)	Overweight (n = 29)	Obesity (n = 22)	*p*-Value
Age (years)	43.45 ± 11.98	49.88 ± 13.01	47.44 ± 10.09	0.163
BMI (kg/m^2^)	22.51 ± 1.29	27.25 ± 1.22	34.32 ± 3.83	<0.01
25(OH)D (ng/mL)	29.65 ± 6.62	25.63 ± 7.02	24.24 ± 5.28	0.127
REG3α (ng/mL)	521.49 ± 311.27	646.03 ± 217.09	574.78 ± 212.07	0.226
IL-6 (pg/mL)	1.50 (1.50–2.52)	2.54 (1.75–3.50)	2.25 (1.55–5.23)	0.126
hs-CRP (mg/dL)	0.12 (0.04–0.20)	0.11 (0.03–0.32)	0.19 (0.07–0.55)	0.144
Ferritin (pg/mL)	72.85 (49.23–107.35)	69.93 (30.62–115.50)	115.00 (54.77–180.40)	0.199
β-defensin-2 (pg/mL)	152.50 (144.36–164.75)	149.10 (143.93–170.02)	157.40 (140.38–175.20)	0.976
Presepsin (ng/mL)	3.77 (2.74–5.52)	2.14 (1.52–3.03)	3.20 (2.06–7.84)	0.008
Vitamin D supplementation	35%	32.4%	33.3%	0.980

Data are expressed as mean ± standard deviation or median (interquartile range) for continuous variables and as counts (percentages) for categorical variables. Between-group comparisons were performed using one-way ANOVA or the Kruskal–Wallis test, as appropriate, and the Chi-square test for categorical variables. When the Kruskal–Wallis test indicated a significant overall difference, pairwise comparisons were performed using the Mann–Whitney U test. Abbreviations: BMI: Body Mass index; 25(OH)D: 25-hydroxy-vitamin D; IL-6: interleukin-6; hs-CRP: high-sensitivity C-reactive; REG3α: regenerating islet-derived protein 3 alpha.

**Table 2:** Independent Predictors of Elevated * REG3α.

Variable	Adjusted OR	95% CI	*p*-Value	Effect Size (r)
Vitamin D supplementation	0.26	0.07–0.95	0.041	−0.28
Age	1.0	0.95–1.05	0.917	0.12
IL-6	0.96	0.81–1.15	0.665	−0.02
β-defensin-2	1.0	0.99–1.01	0.621	0.08

* Elevated circulating REG3α concentrations were defined as values above the sample median. The model included vitamin D supplementation status, age, IL-6, and β-defensin-2 as covariates. Abbreviations: IL-6: interleukin-6; OR: Odds Ratio; CI: Confidence Interval; REG3α: regenerating islet-derived protein 3 alpha.

**Table 3:** Correlations Between REG3α and Other Variables.

Variable	ρ	*p*-Value
β-defensin-2	0.43	0.0002
IL-6	0.28	0.022
hs-CRP	0.19	0.06
Ferritin	0.07	0.47
Presepsin	0.05	0.58
25(OH)D	−0.09	0.42

Abbreviations: 25(OH)D: 25-hydroxy-vitamin D; IL-6: interleukin-6; hs-CRP: high-sensitivity C-reactive protein; REG3α: regenerating islet-derived protein 3 alpha.

## Data Availability

The data presented in the study are available on request from the corresponding author. The data are not publicly available due to privacy restrictions of the Greek National Health System.

## Supplementary Materials

The following supporting information can be downloaded at: https://www.mdpi.com/article/10.3390/cimb47120970/s1.
