# Supplementary material for: Preliminary Study on Circulating REG3α and Its Associations with Vitamin D Supplementation and Inflammatory Biomarkers in Adults with Overweight and Obesity
Source: Curr Issues Mol Biol. 2025 Nov 21;47(12):970. doi: 10.3390/cimb47120970 (PMC12731701; doi:10.3390/cimb47120970)
Supplement: Supplementary file 1 [file cimb-47-00970-s001.zip › cimb-3970069-supplementary.pdf]

Supplementary Table S1. Descriptive and inferential statistics for circulating REG3α across BMI categories

Comparison	Mann–Whitney U	p-value (MW)	Odds Ratio (OR)	95% CI	p-value (Logit)	Cohen's d	Kruskal–Wallis H	p-value (KW)
Overweight vs Control	147.5	0.013	5.73	1.50–21.89	0.011	0.485	6.08	0.048
Obesity vs Control	151.5	0.211	4.20	1.04–16.90	0.043	0.204	—	—
Obesity vs Overweight	377.5	0.270	0.73	0.24–2.26	0.589	0.331	—	—

Abbreviations: BMI – body-mass index; REG3α – regenerating islet-derived protein 3 alpha; CI – confidence interval; OR – odds ratio; MW – Mann–Whitney; KW – Kruskal–Wallis.

Supplementary Figure S1. Histogram showing the distribution of serum REG3α concentrations among all study participants.

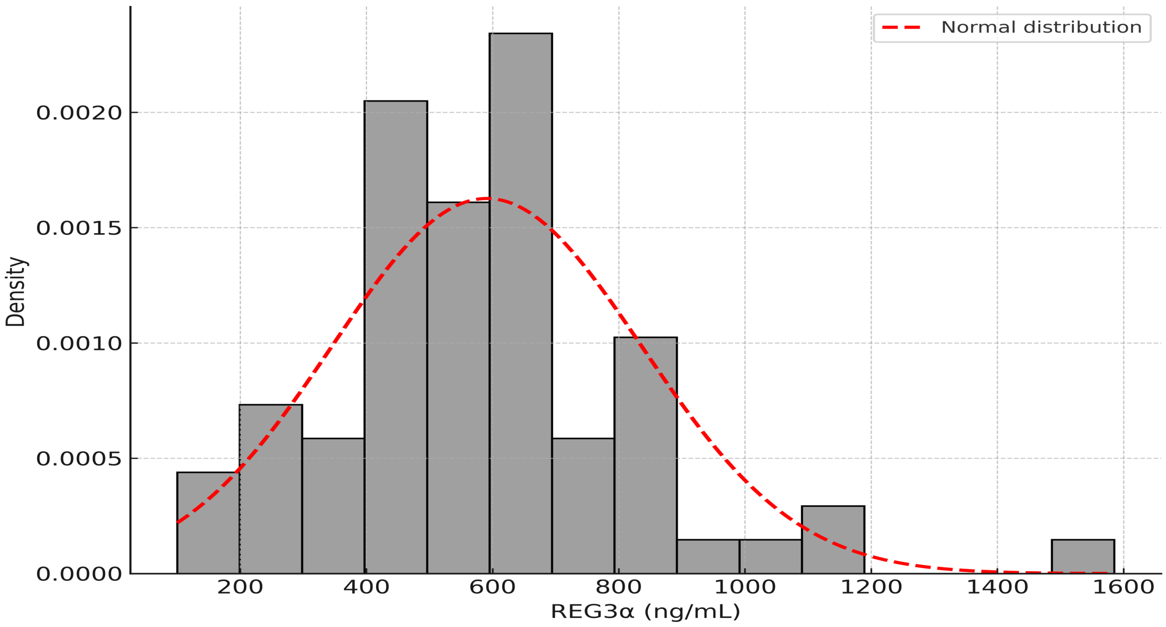

Abbreviations: REG3α – regenerating islet-derived protein 3 alpha.

Supplementary Figure S2. Scatter plots illustrating the relationships between serum REG3α concentrations and 25(OH)D, IL-6, β-defensin-2, hs-CRP, ferritin, and presepsin

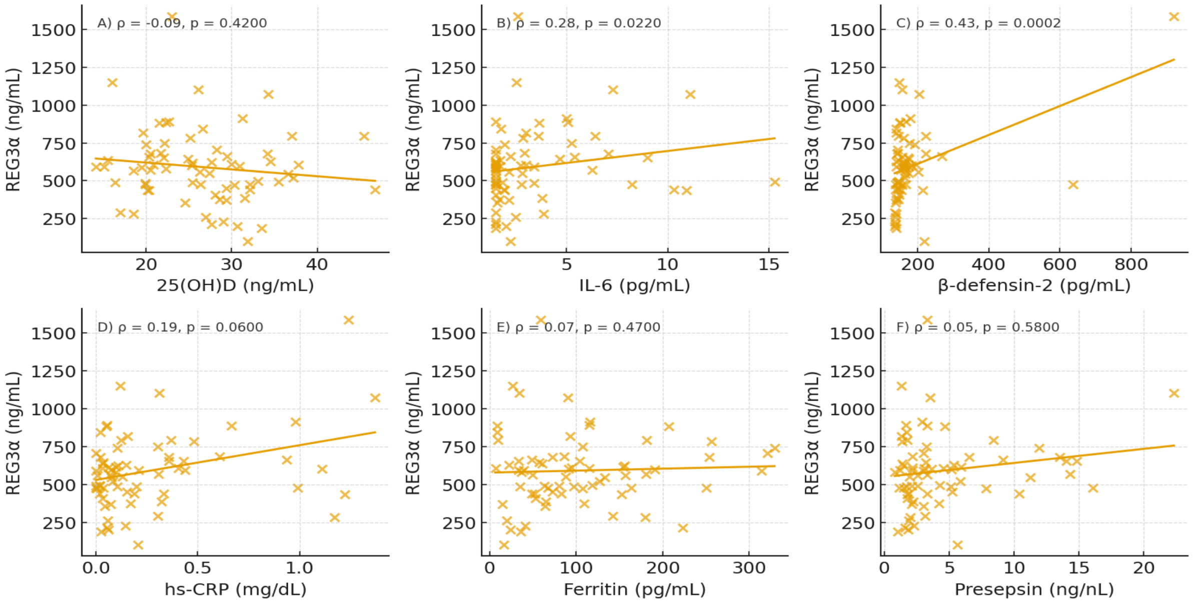

Abbreviations: REG3α – regenerating islet-derived protein 3 alpha; 25(OH)D – 25-hydroxy-vitamin D; IL-6 – interleukin-6; hs-CRP – high-sensitivity C-reactive protein.

Supplementary Figure S3. Principal component analysis of inflammatory and mucosal biomarkers

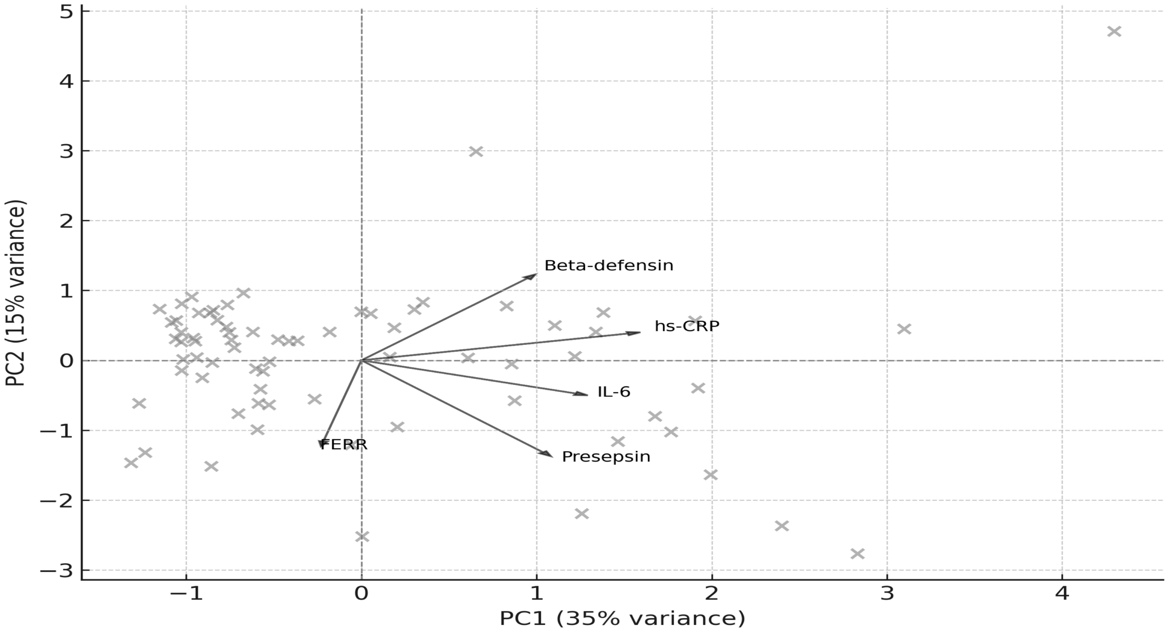

Principal component analysis (PCA) was performed on inflammatory and mucosal biomarkers (IL-6, β-defensin-2, hs-CRP, ferritin, and presepsin) to summarize shared variability. The biplot illustrates the first two principal components (PC1 and PC2), which together explain approximately 50% of the total variance (PC1: ~35%, PC2: ~15%). Biomarker loadings are represented as vectors, indicating their contribution to each component. IL-6 and β-defensin-2 exhibited the strongest loadings on PC1, defining the primary inflammatory–mucosal activation axis. REG3α correlated positively with PC1 ($\varrho = 0.37$, $p = 0.006$), consistent with this inflammatory–mucosal component. **Abbreviations:** PCA – principal component analysis; PC – principal component; REG3α – regenerating islet-derived protein 3 alpha; IL-6 – interleukin-6; hs-CRP – high-sensitivity C-reactive protein; Ferr - ferritin
